# Multiple-level biomedical event trigger recognition with transfer learning

**DOI:** 10.1186/s12859-019-3030-z

**Published:** 2019-09-06

**Authors:** Yifei Chen

**Affiliations:** School of Information Engineering, Nanjing Audit University, 86 West Yushan Road, Nanjing, China

**Keywords:** Event trigger recognition, Transfer learning, Neural networks

## Abstract

**Background:**

Automatic extraction of biomedical events from literature is an important task in the understanding biological systems, allowing for faster update of the latest discoveries automatically. Detecting trigger words which indicate events is a critical step in the process of event extraction, because following steps depend on the recognized triggers. The task in this study is to identify event triggers from the literature across multiple levels of biological organization. In order to achieve high performances, the machine learning based approaches, such as neural networks, must be trained on a dataset with plentiful annotations. However, annotations might be difficult to obtain on the multiple levels, and annotated resources have so far mainly focused on the relations and processes at the molecular level. In this work, we aim to apply transfer learning for multiple-level trigger recognition, in which a source dataset with sufficient annotations on the molecular level is utilized to improve performance on a target domain with insufficient annotations and more trigger types.

**Results:**

We propose a generalized cross-domain neural network transfer learning architecture and approach, which can share as much knowledge as possible between the source and target domains, especially when their label sets overlap. In the experiments, MLEE corpus is used to train and test the proposed model to recognize the multiple-level triggers as a target dataset. Two different corpora having the varying degrees of overlapping labels with MLEE from the BioNLP’09 and BioNLP’11 Shared Tasks are used as source datasets, respectively. Regardless of the degree of overlap, our proposed approach achieves recognition improvement. Moreover, its performance exceeds previously reported results of other leading systems on the same MLEE corpus.

**Conclusions:**

The proposed transfer learning method can further improve the performance compared with the traditional method, when the labels of the source and target datasets overlap. The most essential reason is that our approach has changed the way parameters are shared. The vertical sharing replaces the horizontal sharing, which brings more sharable parameters. Hence, these more shared parameters between networks improve the performance and generalization of the model on the target domain effectively.

## Background

Recently, as interest in biomedical research grows, an overwhelming amount of literature has been published online. As a result, there are incremental studies in applying Text Mining (TM) techniques for automatic recognizing and tracking of the new discoveries and theories in these biomedical articles. These biomedical TM applications include named entity (e.g. gene and protein mentions) recognition, relation (e.g. protein-protein interactions) extraction between entities, and event (e.g. gene transcriptions and regulations) extraction, etc [1–3].

Event extraction refers to automatically extracting structured representations of biomedical relations, functions and processes from text [3]. Since the BioNLP’09 [4] and BioNLP’11 [5] Shared Tasks, event extraction has become a research focus. The structure of each event is defined as an arbitrary number of participants to indicate functions and processes on molecular level, such as “regulation” and “phosphorylation”. When a certain protein regulates the expression of a certain gene and its products are in turn involved in some phosphorylation processes, the “regulation” and “phosphorylation” events come into being. Event extraction task usually contains two main steps: identifying the event triggers and then identifying the event arguments according to the triggers [6]. Event trigger recognition, aiming at detecting those expressions from text that indicate certain events, is the first and crucial step of event extraction. Event extraction performance depends entirely on the recognized triggers. This point was clearly shown by Bj$\ddot {o}$rne et al. [7]. They found that between using the gold standard and predicted triggers, the performance declined by more than 20 points. Many Machine Learning (ML) based methods, including Conditional Random Field (CRF) [8, 9], Support Vector Machine (SVM) [7, 10–13], and Deep Neural Network (DNN) [14–16] models have been successfully applied to event trigger recognition.

These machine learning based approaches rely on large quantity and high quality annotated training data. Their performance may deteriorate when certain training instances are insufficient. However, acquiring manually annotated datasets is both time consuming and costly. Up to now, the manual annotations of biological events mainly focus on genes and proteins. In the corpora of the Shared Tasks of BioNLP’09, 9 types of frequently used biomolecular events are annotated. Biomolecular events involving proteins and genes are an important part of the picture of biological systems, but still only a small part. Hence, in order to obtain a more comprehensive understanding of biological systems, the scope of event extraction has been broadened from molecular-level reactions to cellular-, tissue- and organ-level effects, and to organism-level outcomes [17]. It is not trivial to keep up to date with the annotations of the expanding event types across multiple levels. For example, in the MLEE corpus [10] multiple levels of events from the molecular level to the whole organism have been annotated. The number of event types has been extended to 19. But at the same time, the number of annotated instances for each event type has been greatly reduced. Thus, it will be useful that the annotated dataset from a related domain (such as biomolecular event annotations from the BioNLP’09 corpus) can help to alleviate the shortage of training data problem in the target domain (such as multiple-level event recognition from the MLEE corpus). Recently, transfer learning (TL) techniques have been proposed to address this need [18].

The concept of transfer learning comes from the observed fact that when learning in a new related domain, humans can usually benefit from what they have learned before [19]. This idea has been employed in data mining and machine learning fields [20–22] as a transfer learning schema. Pan and Yang [18] define transfer learning as using some knowledge learned from a source dataset to perform a task on a target dataset. And, transfer learning has been successfully applied to many fields, including text mining [23, 24].

Here, we focus on the research of transfer learning for DNNs, due to their successful application in many text mining tasks over the last few years. Ideally, transfer learning can achieve higher performance by reducing the amount of annotated data needed, and improving generalization of the model on the target dataset. Normally, in the setting of TM and Natural Language Processing (NLP), according to the difference between the source and target datasets, transfer learning approaches of DNN models have three common categories: cross-lingual transfer, cross-domain transfer and cross-task transfer. Due to different languages, cross-lingual transfer is mostly limited to the use of additional language resources to transfer knowledge [25, 26] between the source and target datasets. It cannot extend to our biomedical event trigger recognition applications across multiple levels.

Sharing the same language, both cross-domain and cross-task transfer learning modes can take advantage of more relevance between source and target datasets. In these two modes, parameters of DNN models are used to transfer knowledge between source and target datasets. Some parameters of one model learned from a source dataset can be converted to initialize some parameters of another related model for optimizing on a target dataset. Usually, how many parameters can be shared depends on the degree of the relevance of the source and target datasets. Yang [27] examined the effects of transfer learning for deep hierarchical recurrent networks on several different sequence labelling tasks, including the cross-domain, cross-task and cross-lingual transfer learning models. And it was reported that significant improvement can be obtained. In the case of cross-domain transfer, the datasets of two domains are consistent when their label sets are identical or mappable to each other. Otherwise, the datasets of two domains are inconsistent. If the two domains are consistent, they can share the parameters of all the layers between the source and target DNN models. But, if they are inconsistent, the parameter sharing is restricted to the fewer layers of the DNN models. The cross-task transfer can be simply considered as the case of the cross-domain transfer using inconsistent label sets due to the fact that different tasks do not share the same tags. Hence, the same parameter sharing strategy is effective for them [27]. In the work of Meftah [28], both cross-task and cross-domain (with inconsistent source and target tags) transfer learning was implemented to address the problem of the need in annotated data of social media texts. And the validity and genericity of the models were demonstrated on the Part-Of-Speech (POS) tagging tasks. More studies on transfer learning have been successfully performed in the NLP sequence labelling tasks. Dong [29] proposed a multichannel DNN model to transfer knowledge cross-domain in Chinese social media. In order to ensure the consistency of the source and target domains, some tags are merged in their paper. The experiments showed that the model achieved the best advanced performance. Lee [24] used cross-domain transfer learning for Named Entity Recognition (NER) with consistent tags, showing that transfer learning improved upon the state-of-the-art results on a target dataset with a small number of instances. Giorgi [30] demonstrated that transferring a DNN model significantly improved the latest leading results for biomedical NER, when the source and target domains are consistent.

Our aim in this study is to transfer the trigger recognition knowledge from the source molecular level domain to the target multiple-level domain. This can be seen as an exploratory step towards the more effective automatic extraction of targets from a complex and multifarious domain based on an available simple and singular domain. This situation often occurs in certain fields when research is extended from a familiar area to an unfamiliar and broader area. For instance, after the 9 types of molecular level event relationships between genes and proteins from the biomedical literature have been studies, the research focus will shift to other levels, and the event types will be expanded. The source and target domains, event triggers from different levels, are highly related. Under this circumstance, their label sets may overlap more or less. Nevertheless the annotations from the source and target domains are inconsistent, because their label sets are not identical and mappable. However, among all the above transfer learning studies, there is no model designed to solve how to share network parameters in the case of overlapping label sets. They just simplify the problem to the case of having different label sets between the source and target domains.

We present a new generalized transfer learning approach based on a DNN model, which attempts to share the knowledge to the extent possible between the related source and target domains. The transfer learning approach is modified and generalized to share more network parameters to improve trigger recognition performance across multiple levels on the target domain. Our approach mainly addresses transfer learning between the domains with overlapping label sets. In this paper, a source domain with plentiful annotations of biomolecular event triggers (the BioNLP corpus) is used to improve performance on a target domain of multiple-level event triggers with fewer available annotations (the MLEE corpus). To our knowledge, no reported research has applied transfer learning to make the best use of overlapping label sets to find the shared knowledge.

The rest of this paper is organized as follows. In “Methods” section, detailed descriptions of the proposed generalized transfer learning method and Multiple-Level Trigger recogNizer (MLTrigNer) system are provided. “Results” section describes the used biomedical corpora, experimental settings, and all the experimental results. And this is followed by the in-depth analysis in “Discussion” section. We present the conclusions and future work in “Conclusions” section.

## Results

### Corpus description

An in-depth investigation is carried out to compare the performance of our proposed Multiple-Level event Trigger recogNizer, MLTrigNer, which is built based on the generalized cross-domain transfer learning BiLSTM-CRF model. The dataset *Data*_*MLEE*_ is used as the target domain dataset. With varying degrees of label overlapping, *Data*_*ST*09_ and *Data*_*EPI*11_ are used as the source domain datasets, respectively. Named entity and trigger types annotated in these corpora are illustrated in Table 1. In the trigger types of *Data*_*MLEE*_, the labels overlapped with *Data*_*ST*09_ are marked using ‘*’, and the labels overlapped with *Data*_*EPI*11_ are marked using ‘+’. We can see that *Data*_*MLEE*_ and *Data*_*ST*09_ are highly related because of the nine overlapping trigger labels. However, there are some overlapping labels that have gone beyond the molecular level in *Data*_*MLEE*_, which annotate events across multiple levels. For example, “Localization” is the event type extracted from both cells and biomolecules in *Data*_*MLEE*_. *Data*_*MLEE*_ and *Data*_*EPI*11_ are loosely related with only two overlapping trigger labels. More details of these datasets are introduced in the following.

**Table 1:** Named entity and trigger types in *Data*_*MLEE*_, *Data*_*ST*09_ and *Data*_*EPI*11_, respectively

Corpus	Named entity type	Trigger type
*Data*_*ST*09_	Protein	Gene expression*
		Transcription*, Binding*
		Protein catabolism*
		Phosphorylation*
		Localization*, Regulation*
		Positive regulation*
		Negative regulation*
*Data*_*EPI*11_	Protein	Hydroxylation, Dehydroxylation
		Phosphorylation+, Deglycosylation
		Dephosphorylation+, Catalysis
		Ubiquitination, Acetylation
		Deubiquitination
		DNA methylation
		DNA demethylation
		Glycosylation, Deacetylation
		Methylation, Demethylation
*Data*_*MLEE*_	Gene or gene product	Cell proliferation, Planned process
	Drug or compound	Development, Binding*
	Developing anatomical structure	Blood vessel develop
	Organ, Tissue	Growth, Death, Regulation*
	Immaterial anatomical entity	Breakdown, Remodeling
	Anatomical system	Synthesis, Localization*
	Organism, Cell	Gene expression*
	Pathological formation	Transcription*
	Organism subdivision	Protein catabolism*
	Multi-tissue structure	Phosphorylation*+
	Cellular component	Dephosphorylation+
	Organism substance	Positive regulation*
		Negative regulation*

In the trigger types of *Data*_*MLEE*_, the labels overlapped with *Data*_*ST*09_ are marked using ‘*’, and the labels overlapped with *Data*_*EPI*11_ are marked using ‘+’

#### *Data*_*MLEE*_

The MLEE corpus [10] is used to train and test our MLTrigNer on multiple-level trigger word identification as a target dataset. The corpus is taken from 262 PubMed abstracts focusing on tissue-level and organ-level processes, which are highly related to certain organism-level pathologies. In *Data*_*MLEE*_, 19 event types are chosen from the GENIA ontology, which can be classified into four groups: anatomical, molecular, general and planned. Our task is to identify the correct trigger type of each event. Hence, there are 20 tags in the target label set, including a negative one. All the statistics in the training, development and test sets are shown in Table 2.

**Table 2:** Statistics of documents, words and events in the dataset *Data*_*MLEE*_, including the training set, the development set, and the test set, respectively

Item	Training	Development	Test
Document	131	44	87
Words	27,875	9610	19,103
Event	3296	1175	2206

#### *Data*_*ST*09_

This corpus is taken from the Shared Task (ST) of BioNLP challenge 2009 [4] and contains training and development sets, including 950 abstracts from PubMed. It is used to train our MLTrigNer as a source dataset. In this corpus, 9 event types are chosen from the GENIA ontology involving molecular-level entities and processes, which can be categorized into 3 different groups: simple events, binding events and regulation events. The training and development sets are combined as a source domain dataset *Data*_*ST*09_. All of the detailed statistics of *Data*_*ST*09_ are shown in Table 3.

**Table 3:** Statistics of documents, words and events in the training set, the development set and their combination as *Data*_*ST*09_, respectively

Item	Training	Development	*Data*_*ST*09_
Abstract	800	150	950
Words	176,146	33,937	210,083
Event	8597	1809	10,406

#### *Data*_*EPI*11_

This corpus is taken from the Epigenetics and Post-translational Modifications (EPI) task of BioNLP challenge 2011 [5] and contains training and development sets, including 800 abstracts relating primarily to protein modifications drawn from PubMed. It is also used to train our MLTrigNer as a source dataset. In this corpus, 14 protein entity modification event types and their catalysis are chosen. Hence there are 15 event types totally. The training and development sets are combined as a source domain dataset *Data*_*EPI*11_. All of the detailed statistics in *Data*_*EPI*11_ are shown in Table 4. The number of annotated events in *Data*_*EPI*11_ is less than that in the *Data*_*ST*09_, annotating the more event types.

**Table 4:** Statistics of documents, words and events in the training set, the development set and their combination as *Data*_*EPI*11_, respectively

Item	Training	Development	*Data*_*EPI*11_
Abstract	600	200	800
Words	127,312	43,497	170,809
Event	1852	601	2453

### Performance assessment

We measure the performance of the trigger recognition system in terms of the *F*1 measure. The *F*1 is determined by a combination of precision and recall. Precision is the ratio of the number of correctly classified triggers within a category to the total number of recognized ones. Recall is the ratio of the number of correctly classified triggers within a category to the total number of triggers. They are defined as follows:

1 $$ F1-measure = \frac{2Precision \times Recall}{Precision+Recall}  $$

2 $$ Precision = \frac{TP}{TP+FP}  $$

3 $$ Recall = \frac{TP}{TP+FN}  $$

where *TP* is the number of the triggers that are correctly classified to a category, *FP* is the number of the triggers that are misclassified to a category, and *FN* is the number of the triggers misclassified to other categories.

### Implementation details

All of the experiments described in the following are implemented using the Tensorflow library [31]. Hyperparameters are tuned using the training and development sets through cross-validation and then the final model is trained on the combined set of the optimal ones. We tune the pre-trained word embedding vector *E*^*w*^ to 200 dimensions, character embedding vector *E*^*c*^ to 100 dimensions, named entity type embedding vector *E*^*e*^ to 10 for source domain while 50 dimensions for target domain, POS embedding vector *E*^*p*^ to 50 dimensions, pre-trained dependency tree-based word embedding vector *E*^*d*^ to 300 dimensions. Then, the BiLSTM layer with a hidden state dimension of 300, and the fully-connected layer with 600 dimensions. In order to avoid overfitting, dropout with a probability 0.5 is used before the input to the BiLSTM and fully-connected layers.

### Transfer learning performance

**Fig. 1:**
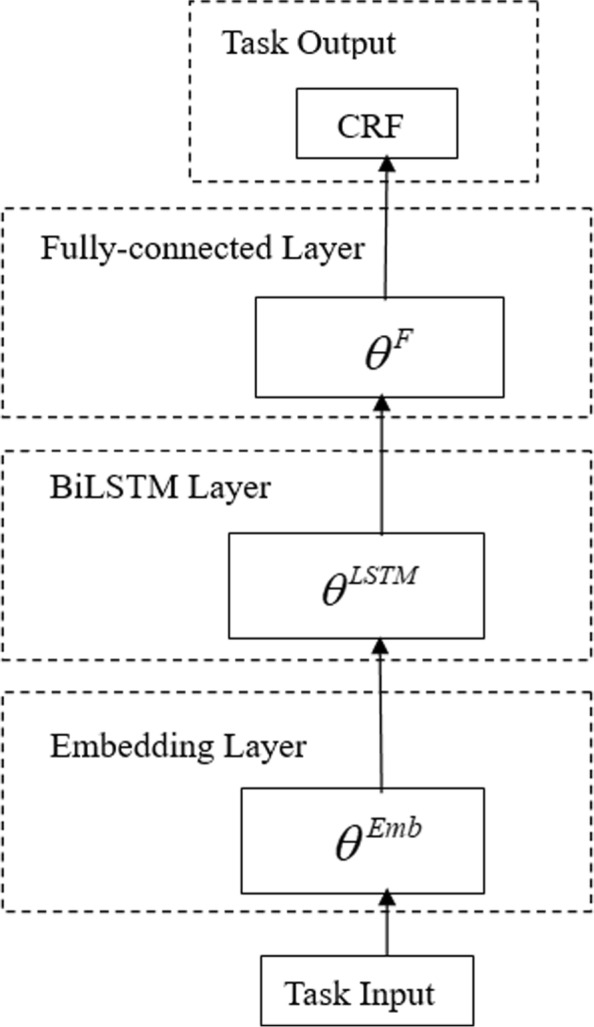
The network architecture of Basic Model A: the BiLSTM-CRF model, having a Embedding layer, a BiLSTM layer, a Fully-connected layer and a CRF layer

**Fig. 2:**
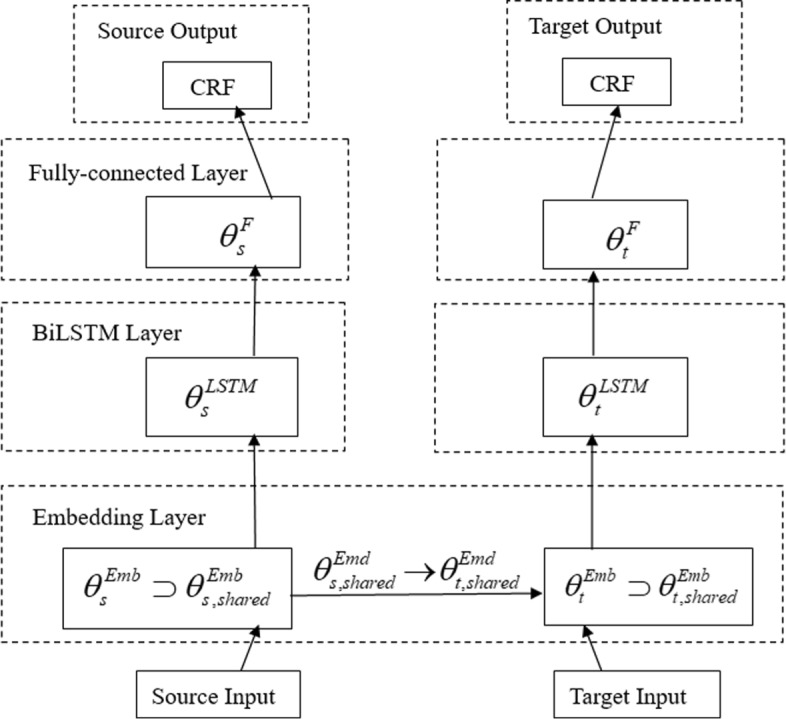
The network architecture of TL Model C: Transfer learning BiLSTM-CRF model with the different feature and label sets, having Embedding layers, BiLSTM layers, Fully-connected layers and CRF layers for the source and target networks, respectively. The parameters can be transferred in the Embedding layers only

**Fig. 3:**
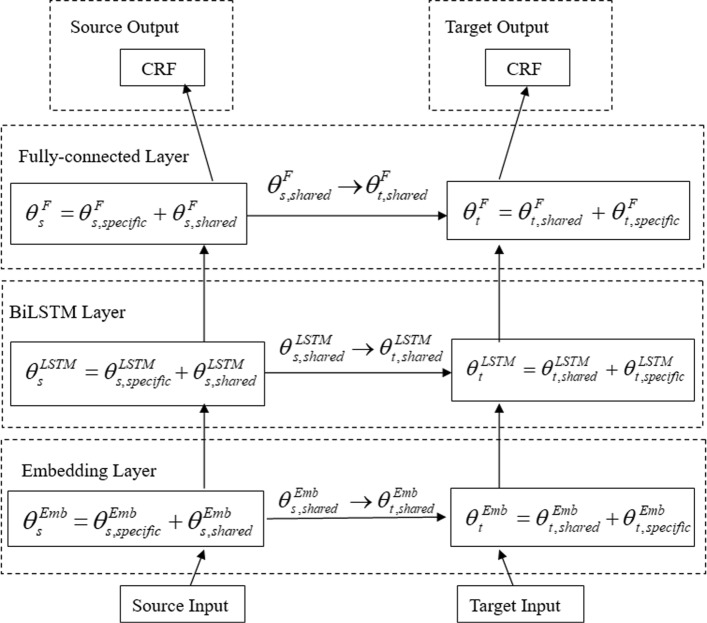
The network architecture of Generalized TL Model D: Our proposed generalized transfer learning BiLSTM-CRF model for Multiple-Level Trigger recogNizer, MLTrigNer. It has Embedding layers, BiLSTM layers, Fully-connected layers and CRF layers for the source and target networks, respectively. The parameters can be transferred in all the Embedding layers, the BiLSTM layers and Fully-connected layers

**Fig. 4:**
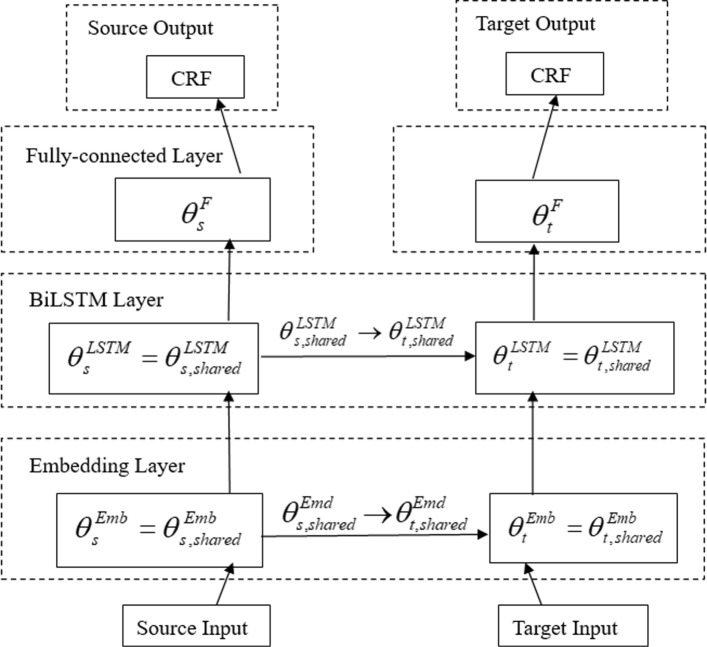
The network architecture of TL Model B: Transfer learning BiLSTM-CRF model with the different label sets, having Embedding layers, BiLSTM layers, Fully-connected layers and CRF layers for the source and target networks, respectively. The parameters can be transferred in the Embedding layers and the BiLSTM layers

The effectiveness of our proposed is approach illustrated based on the performance comparison of the three neural network models described in “Methods” section. First, the Basic Model A (Fig. 1) is trained only on the training and development sets of *Data*_*MLEE*_ (without transfer learning) as a baseline measurement, and its results are shown in the second column of Table 5. Then, *Data*_*ST*09_ is used as the source dataset in the transfer learning models. The TL Model C (Fig. 2) and the MLTrigNer model (Fig. 3) are jointly trained on *Data*_*ST*09_ and the training and development sets of the target dataset *Data*_*MLEE*_ using different transfer learning approaches, respectively. The three models are tested on the test set of *Data*_*MLEE*_. The results are shown in the third and forth columns of Table 5. Among the models described in “Methods” section, the TL Model B (Fig. 4) cannot be used in the trigger recognition task since the domain-dependent input feature sets are employed, which are inconsistent in the source and target domains.

**Table 5:** Detailed results achieved by the proposed MLTrigNer Model, Basic Model A and TL Model C on *Data*_*MLEE*_

Trigger type	Basic Model A			TL Model C			MLTrigNer Model		
	P	R	F1	P	R	F1	P	R	F1
Cell proliferation	85.37	81.40	83.33	80.95	79.07	80.00	81.40	81.40	81.40
Development	66.37	76.53	71.09	76.29	75.51	75.90	78.00	79.59	**78.79**
Blood vessel develop	97.33	94.19	95.74	97.98	93.87	95.88	100.0	94.52	**97.18**
Growth	96.00	85.71	90.57	90.74	87.50	89.09	92.31	85.71	88.89
Death	73.68	75.68	74.67	67.39	83.78	74.70	69.57	86.49	**77.11**
Breakdown	82.35	63.64	71.79	75.00	68.18	71.43	87.50	63.64	**73.68**
Remodeling	71.43	50.00	58.82	55.55	50.00	52.63	85.71	66.67	**75.00**
Synthesis	50.00	25.00	33.33	0.0	0.0	0.0	100.0	100.0	**100.0**
Gene expression	91.67	83.33	87.30	91.80	84.85	88.19	94.44	90.15	**92.25**
Transcription	0.0	0.0	0.0	50.00	16.67	25.00	100.0	66.67	**80.00**
Protein Catabolism	0.0	0.0	0.0	0.0	0.0	0.0	100.0	60.00	**75.00**
Phosphorylation	75.00	100.0	85.71	60.00	100.0	75.00	75.00	100.0	85.71
Dephosphorylation	0.0	0.0	0.0	0.0	0.0	0.0	0.0	0.0	0.0
Localization	78.83	81.20	80.00	82.09	82.71	82.40	82.17	79.70	80.92
Binding	86.96	70.18	77.67	83.02	77.19	80.00	91.49	75.43	**82.69**
Regulation	59.80	58.93	59.37	60.27	63.77	61.97	61.71	66.18	**63.87**
Positive regulation	80.88	81.90	81.39	84.82	81.59	83.17	84.81	85.07	**84.94**
Negative regulation	84.73	65.71	74.02	80.75	78.78	79.75	78.96	75.10	76.99
Planned process	78.69	48.98	60.38	74.15	55.61	63.56	78.76	58.67	67.25
TOTAL	81.63	74.26	77.77	81.52	77.66	79.53	83.31	79.40	**81.31**

The Basic Model A is trained only on the training and development sets of *Data*_*MLEE*_ without transfer learning. The TL Model C and the MLTrigNer model are jointly trained on the source dataset *Data*_*ST*09_ and the training and development sets of the target dataset *Data*_*MLEE*_ using different transfer learning approaches, respectively. The three models are tested on the test set of *Data*_*MLEE*_. In the results of MLTrigNer Model, the improved F1 values are marked in bold

From the results of the Basic Models A and the TL Model C, we can see that the transfer learning improves the *F*1 measure 1.76%. Generalizing the transfer learning schema in the MLTrigNer Model improves the trigger recognition performance a further 1.78%. This improvement is due to the fact that in our approach, more parameters are transferred from the source network to the target one than usual, signifying more effective knowledge sharing. It is worth noting there are improvements in both precision and recall, which refers to the ability of the MLTrigNer to identify more positive triggers. Higher precision and recall signify identification of more potential biomedical events during the subsequent processing phase, which is important for the ultimate event extraction application. Compared with the TL Model C, beside “Negative regulation” and “Localization”, the *F*1 values of the other trigger types overlapping with the source dataset are improved. Among these overlapping labels, some of them have gone beyond the molecular level in *Data*_*MLEE*_ to annotate events across multiple levels. Moreover, the *F*1 values of the 7 non-overlapping trigger types are also improved, except for “Growth”, “Dephosphorylation” and “Planned process”. Hence, our proposed approach can improve the recognition performance across multiple levels through transfer more knowledge from a single level domain.

Then, *Data*_*EPI*11_ is used as the source dataset alternatively. Basic Model A (Fig. 1) was also trained only on the training and development sets of *Data*_*MLEE*_ (without transfer learning) as a baseline measurement, and its results are shown in the second column of Table 6. The TL Model C (Fig. 2) and the MLTrigNer Model (Fig. 3) are then jointly trained on the source dataset *Data*_*EPI*11_ and the training and development sets of the target dataset *Data*_*MLEE*_ using different transfer learning approaches. The results are shown in the third and forth columns of Table 6, respectively. The three models are tested on the test set of *Data*_*MLEE*_.

**Table 6:** Detailed results achieved by the proposed MLTrigNer Model, Basic Model A and TL Model C on *Data*_*MLEE*_

Trigger type	Basic Model A			TL Model C			MLTrigNer Model		
	P	R	F1	P	R	F1	P	R	F1
Cell proliferation	85.37	81.40	83.33	83.33	81.40	82.35	81.40	81.40	81.40
Development	66.37	76.53	71.09	74.51	77.55	76.00	78.35	77.55	**77.95**
Blood vessel develop	97.33	94.19	95.74	98.64	93.87	96.20	98.99	94.84	**96.87**
Growth	96.00	85.71	90.57	88.89	85.71	87.27	92.45	87.50	89.91
Death	73.68	75.68	74.67	66.67	81.08	73.17	66.67	81.08	73.17
Breakdown	82.35	63.64	71.79	73.68	63.64	68.29	87.50	63.64	**73.68**
Remodeling	71.43	50.00	58.82	75.00	30.00	42.86	66.67	40.00	50.00
Synthesis	50.00	25.00	33.33	33.33	25.00	28.57	20.00	25.00	22.22
Gene expression	91.67	83.33	87.30	85.51	89.39	87.41	89.05	92.42	**90.71**
Transcription	0.0	0.0	0.0	50.00	16.67	25.00	100.0	16.67	**28.57**
Protein Catabolism	0.0	0.0	0.0	0.0	0.0	0.0	33.33	20.00	**25.00**
Phosphorylation	75.00	100.0	85.71	100.0	100.0	100.0	100.0	100.0	100.0
Dephosphorylation	0.0	0.0	0.0	0.0	0.0	0.0	0.0	0.0	0.0
Localization	78.83	81.20	80.00	77.14	81.20	79.12	82.17	79.70	**80.92**
Binding	86.96	70.18	77.67	83.02	77.19	80.00	79.25	73.68	76.36
Regulation	59.80	58.93	59.37	65.13	61.65	63.34	65.17	63.29	**64.22**
Positive regulation	80.88	81.90	81.39	81.11	82.91	82.00	81.96	82.22	**82.09**
Negative regulation	84.73	65.71	74.02	77.18	75.61	76.39	80.72	73.47	**76.92**
Planned process	78.69	48.98	60.38	66.86	57.65	61.92	71.07	57.65	**63.66**
TOTAL	81.63	74.26	77.77	79.69	77.62	78.64	81.76	77.71	**79.68**

The Basic Model A is trained only on the training and development sets of *Data*_*MLEE*_ without transfer learning. The TL Model C and the MLTrigNer model are jointly trained on the source dataset *Data*_*EPI*11_ and the training and development sets of the target dataset *Data*_*MLEE*_ using different transfer learning approaches, respectively. The three models are tested on the test set of *Data*_*MLEE*_. In the results of MLTrigNer Model, the improved F1 values are marked in bold

From the results of the Basic Model A and the TL Model C, we can see that the transfer learning improves the *F*1 measure 0.87%. The MLTrigNer Model improves the performance a further 1.04%, and the improvements are also both in precision and recall. Using *Data*_*EPI*11_ as the source dataset, the MLTrigNer Model brings less performance improvement. This is due to the decreased correlation between the source and target domains. In the transfer learning models, less parameters can be transferred from the source to the target networks. However, our MLTrigNer Model still can improve the performance further compared with the basic transfer learning approach. Hence, our proposed method is effective when the overlapping is more or less. Compared with the TL Model C, the recognition performance of the overlapping trigger “Phosphorylation” is not improved, and its F1 measure is 100.0 in both models, which cannot be improved further. Moreover, the performance of the 13 non-overlapping trigger types are all improved.

### MLTrigNer compared with other trigger recognition systems

We compare the performance of the proposed transfer learning based trigger recognition system, MLTrigNer, with other leading systems on the same *Data*_*NMLEE*_ dataset. Since *Data*_*ST*09_ as the source dataset shows the better performance from the results in Tables 5 and 6, we utilized *Data*_*ST*09_ to train the MLTrigNer Model as the source dataset. The detailed *F*1 measure results are illustrated in Table 7.

**Table 7:** Detailed performance results achieved by the proposed MLTrigNer and the other leading trigger recognition systems, respectively

Trigger Recognition System	Precision	Recall	F1-Measure
Our MLTrigNer system	83.31	79.40	**81.31**
SVM-based System [10]	81.44	69.48	75.67
SVM-based System [13]	75.56	81.29	78.32
Neural Network based System [14]	71.04	84.60	77.23
CNN-based System [15]	80.67	76.76	78.67
RNN-based System [16]	79.78	78.45	79.11

In these results, the best F1 value of our MLTrigNer system is marked in bold

Pyysalo et al. [10] defined an SVM-based classifier with rich hand-crafted features to recognize triggers in the text. Zhou et al. [13] also defined an SVM-based classifier with word embeddings and hand-crafted features. Nie et al. [14] proposed a word embedding-assisted neural network model to model semantic and syntactic information in event trigger identification (the results were converted to 19 categories). Wang et al. [15] defined a window-based convolution neural network (CNN) classifier. Rahul et al. [16] proposed a method that uses a recurrent neural network (RNN) to extract higher-level sentence features in trigger identification.

From Table 7, we can draw two conclusions. First, our generalized transfer learning approach achieves the best result on the dataset *Data*_*MLEE*_, which indicates that our MLTrigNer can still improve the performance of biomedical trigger word recognition. Second, from Table 5, the TL Model C achieves competitive results compared to these leading systems, which means that the improvement of our generalized transfer learning approach is achieved on a relatively strong basis.

## Discussion

### Transfer performance analysis on highly related domains

We conduct an in-depth study and detailed comparison on the highly related domains of *Data*_*ST*09_ and *Data*_*MLEE*_ to show the learning ability of our proposed approach. In our study, there are two datasets with the different overlapping degrees of the labels used as source domains to transfer knowledge, respectively. Between them, *Data*_*ST*09_ is highly related with the target domain. Its trigger types are nested in those of the target domain dataset from Table 1. Hence, we can simply put the *Data*_*ST*09_ and the training and development sets of *Data*_*MLEE*_ together to train the BiLSTM-CRF model without transfer learning (Basic Model A), and then the model is tested on the test set of *Data*_*MLEE*_. Its performance is shown in Table 8 in the line of “Basic Model A (*Data*_*MLEE*_ + *Data*_*ST*09_)”. For the purpose of comparison, in the line of “Basic Model A (*Data*_*MLEE*_)”, the performance of Basic Model A trained on the training and development sets of *Data*_*MLEE*_ and tested on the test set of *Data*_*MLEE*_ is listed. And in the last line, the performance of our MLTrigNer Model is shown, which uses *Data*_*ST*09_ and *Data*_*MLEE*_ as the source and target datasets, respectively. From the results we can see that the performance even declines when just simply mixing nested datasets together. On the other hand, the performance can be improved using our transfer learning approach. In the process of trigger recognition, the shared knowledge brought by the transfer learning is more important than the data itself.

**Table 8:** Detailed performance results on highly related domains with different training modes, including the Basic Model A (trained on the training and development sets of *Data*_*MLEE*_), the Basic Model A (trained on the combination of *Data*_*ST*09_ and the training and development sets of *Data*_*MLEE*_), and our MLTrigNer Model (using *Data*_*MLEE*_ as the target dataset and *Data*_*ST*09_ as the source dataset)

Trigger Recognition System	Precision	Recall	F1-Measure
Basic Model A (*Data*_*MLEE*_)	81.63	74.26	77.77
Basic Model A (*Data*_*MLEE*_ + *Data*_*ST*09_)	78.78	73.92	76.28
Our MLTrigNer Model (*Data*_*MLEE*_ + *Data*_*ST*09_)	83.31	79.40	**81.31**

In these results, the best F1 value of our MLTrigNer model is marked in bold

### Ratio effect analysis on source data

**Fig. 5:**
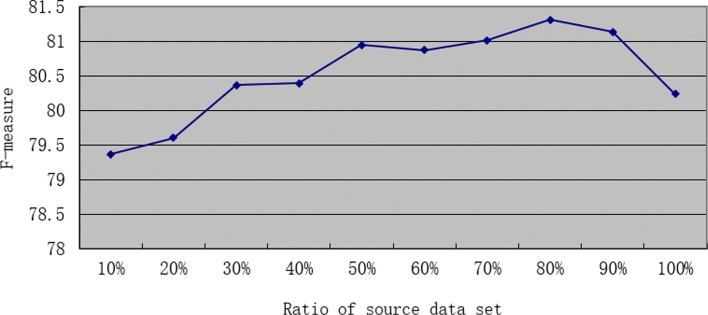
The ratio effect of source domain data *Data*_*ST*09_ to our transfer learning model, MLTrigNer, with the ratio as 10%, 20%, 30%, 40%, 50%, 60%, 70%, 80%, 90% and 100%

It is important to analyze the effect of the ratio of source domain data. First, we use *Data*_*ST*09_ as the source dataset, which is more than 3.6 times the size of the target domain dataset. We keep the size of target data unchanged, and gradually change the size of source data. The changes in the MLTrigNer Model results are shown as a curve in Fig. 5, with the source ratio as 10%, 20%, 30%, 40%, 50%, 60%, 70%, 80%, 90% and 100%. We can see that *F*1 first goes up continuously as the source data is added. Then it reaches a maximum of 81.31 when the source ratio is 80%. Finally, it trends downwards even as more source data is added, reaching 80.46 with 100% data in *Data*_*ST*09_. The results verify that more data from source domain does not always lead to better performance in target domain. In our study, the optimal source/target ratio is about 2.9:1 when maximum performance achieved in *Data*_*MLEE*_. In order to optimize the performance of the model under different datasets, we set the ratio of source domain data to be one of the important hyperparameters of the MLTrigNer model, which is tuned on the training and development sets using cross-validation.

**Fig. 6:**
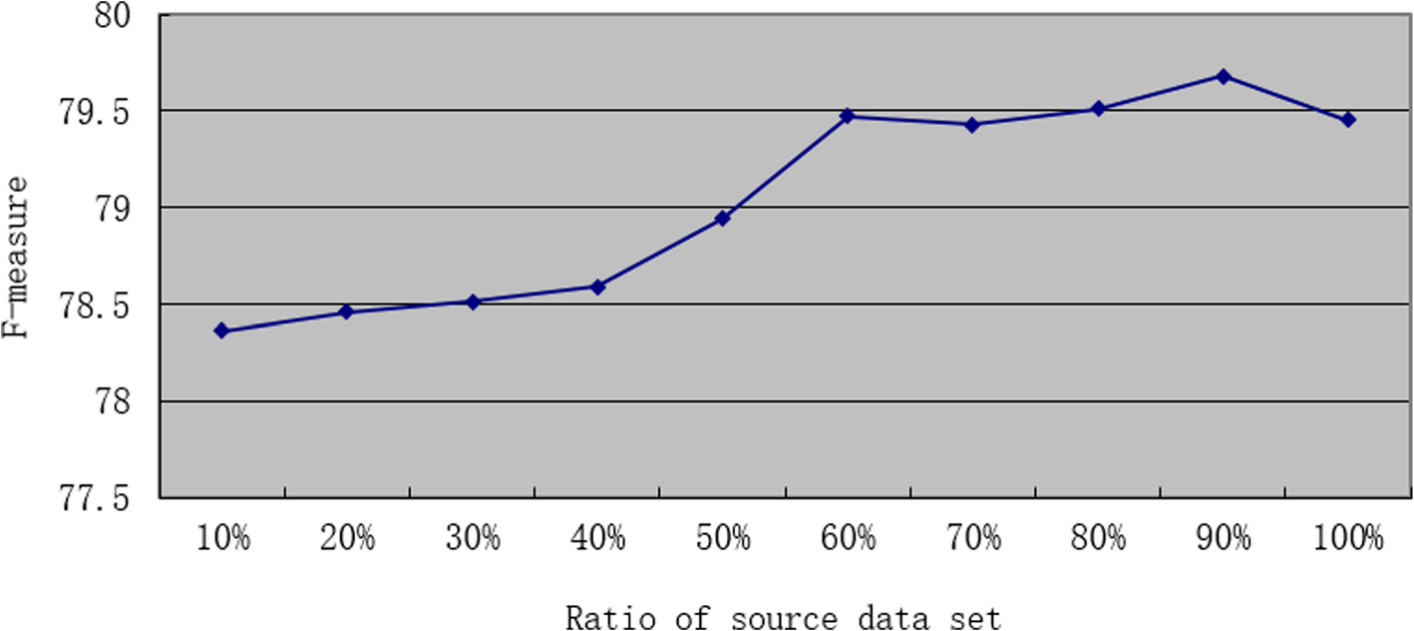
The ratio effect of source domain data *Data*_*EPI*11_ to our transfer learning model, MLTrigNer, with the ratio as 10%, 20%, 30%, 40%, 50%, 60%, 70%, 80%, 90% and 100%

Then, we use *Data*_*EPI*11_ as the source dataset alternatively, which is about 3.1 times the size of the target domain dataset. We also keep the size of the target data unchanged, and gradually change the size of the source data. The changes in the MLTrigNer Model results are shown as a curve in Fig. 6, with the source ratio as 10%, 20%, 30%, 40%, 50%, 60%, 70%, 80%, 90% and 100%. Similar trends are found in the Figs. 5 and 6. The values of *F*1 measure first goes up continuously as source training data is added, and reaches a maximum of 79.68 when the source ratio is 90%. Then, it trends downwards even as more source data is added, reaching 79.45 with 100% data in *Data*_*EPI*11_. After tuned on the training and development sets using cross-validation, the optimal source/target ratio is about 2.7:1 when maximum performance achieved in *Data*_*MLEE*_.

### Error analysis

From the metrics in Tables 5 and 6 we can notice that the results of the trigger type “Dephosphorylation” are all zeroes regardless of the models. From a more detailed list of types and sizes of trigger words of the *Data*_*MLEE*_ in Table 9, we can see that there are only 6 “Dephosphorylation” instances in the *Data*_*MLEE*_. Without adequate training instances, the recognition results of the Basic Model A and TL Model C are very poor. Moreover, with our transfer learning approach, its recognition results of the MLTrigNer model are still zeroes under the situation that “Dephosphorylation” is an overlapping trigger type. This is a limitation of our transfer learning approach that it cannot transfer enough knowledge from other triggers for labelling the rare trigger types.

**Table 9:** List of types and sizes of trigger words in the *Data*_*MLEE*_, where “Dephosphorylation” is a rare trigger type

	Trigger type	size in *Data*_*MLEE*_
Anatomical	Cell proliferation	133
	Development	316
	Blood vessel develop	855
	Growth	169
	Death	97
	Breakdown	69
	Remodeling	33
Molecular	Synthesis	17
	Gene expression	435
	Transcription	37
	Protein catabolism	26
	Phosphorylation	33
	Dephosphorylation	6
General	Localization	450
	Binding	184
	Regulation	773
	Positive regulation	1327
	Negative regulation	921
Planned	Planned process	643

## Conclusions

In this paper we develop a novel transfer learning approach for multiple-level event trigger recognition based on a DNN model. We design a more general transfer learning approach to set the cross-domain transfer, which can share as much knowledge as possible between the source and target datasets, particularly encompassing the case of overlapping label sets. In the experiments, the source datasets having varying degrees of overlapping labels with the target dataset are utilized to verify the effectiveness of our proposed MLTrigNer model. Compared with the basic transfer learning model, our approach improves the performance on the target domain further. Moreover, its performance exceeds other leading trigger recognition systems on the same MLEE corpus. Hence this study contributes to the effective recognition of biomedical trigger words from text across multiple levels. Through analysis, it is found that there are three essential factors mattering to our cross-domain transfer learning approach: the degree of overlapping of the source and target domains; the number of sharable parameters in each layer of a network; and an appropriate size of the source and target datasets. In the future work, more source datasets from different biomedical event levels with varying degrees of overlapping label tags can be utilized together to improve the performance further.

## Methods

In this section, we introduce our proposed transfer learning approach. Our solution for trigger recognition is based on a Bidirectional LSTM-CRF model (BiLSTM-CRF) [32], which uses a deep neural network, Long Short Term Memory (LSTM) [33], to extract higher-level abstract features to train a CRF [34]. We design a transfer learning approach to allow for joint training with a source dataset, which uses an input feature set and a output label set that overlap with the target dataset, respectively.

We first introduce and describe the architecture of the BiLSTM-CRF model as Basic Model A. We then introduce the cross-domain transfer learning BiLSTM-CRF model with inconsistent label sets as TL Model B, and in addiction with inconsistent input feature sets as TL Model C. Finally, our proposed generalized transfer learning model, Generalized TL Model D, is described in detail. The different architectures of the four models are shown in Figs. 1, 4, 2 and 3, respectively.

### Basic model a: biLSTM-CRF model

We present our trigger recognition task based on the BiLSTM-CRF model as Basic Model A, whose architecture is shown in Fig. 1. In Basic Model A, *θ*s denote all the trainable parameters in each network layer. This model detects trigger words and annotates their types, and its performance servers as the baseline. For a given input sentence {*word*_1_,*word*_2_,...,*word*_*n*_}, the aim of trigger recognition is to output a tag sequence {*tag*_1_,*tag*_2_,...,*tag*_*n*_}, where *word*_*i*_ is a word (or a token) in the sentence and *tag*_*i*_ denotes its corresponding type label. The value of *tag*_*i*_ belongs to the label set, which is a biomedical event type or negative if it does not indicate any event. The BiLSTM-CRF model feeds a set of features for an input embedding layer (with parameters *θ*^*Emb*^), extracts higher-level abstract features in subsequence BiLSTM (with parameters *θ*^*LSTM*^) and fully-connected (with parameters *θ*^*F*^) layers, and trains a CRF layer for the final sequence labelling. The main layers of the BiLSTM-CRF model for trigger recognition are described below.

#### Embedding layer

In order to express both syntactic and semantic information in input sentences, besides each word, *word*_*i*_, we also extract other four features from character, POS, named entity type and dependency parse tree. Through lookup tables, the embedding layer converts each input feature into one of the following representation vectors:

1. Word embedding vector *E*^*w*^: Each word in an input sentence is mapped to a word embedding vector, which contains semantic information from its linear contexts. In this paper, we use a pre-trained word lookup table *LT*^*w*^ learned from PubMed articles using the word2vec model [35].
2. Character embedding vector *E*^*c*^: We use an extra LSTM network to extract the orthographic information from the sequence of characters in each input word. Its parameters *LT*^*c*^ are weights and biases of the LSTM, which are initialized randomly and trained to output a character-level embedding vector.
3. POS embedding vector *E*^*p*^: We train a POS lookup table *LT*^*p*^ to extend the word embedding. It maps the POS tag of each word in an input sentence to a POS embedding vector, which extracts syntactic information from the input word. *LT*^*p*^ is initialized randomly and trained to obtain a mapping lookup table.
4. Named entity type embedding vector *E*^*e*^: We train a lookup table *LT*^*e*^ to map named entity type of each word in an input sentence to an embedding vector to extract domain-dependent information. The named entities were provided by the task data. *LT*^*e*^ is initialized randomly and trained to output a mapping lookup table.
5. Dependency tree-based word embedding vector *E*^*d*^: In order to extend features from linear word contexts to non-linear syntactic contexts, each word from an input sentence is mapped to a dependency tree-based word embedding vector, which contains rich non-linear functional and syntactic information. We use a pre-trained word lookup table *LT*^*d*^ learned from English Wikipedia using the skip-gram model [36].

In the embedding layer, trainable parameter set can be expressed as *θ*^*Emb*^={*LT*^*c*^,*LT*^*p*^,*LT*^*e*^}.

#### BiLSTM layer

This layer takes a concatenation of the output embedding vectors of the previous embedding layer as input, $x_{i}=[E_{i}^{w};E_{i}^{c};E_{i}^{p};E_{i}^{e};E_{i}^{d}]$. Due to the ability to learn long-distance dependencies in a sequence through designed memory cells, LSTM is a powerful tool for sequence labelling tasks [33]. Suppose that an input sequence to a LSTM layer is {*x*_1_,*x*_2_,...,*x*_*T*_}, and it yields an output sequence of {*h*_1_,*h*_2_,...,*h*_*T*_} by employing the following implementation strategy during training [32], where both sequences have the same length *T*:

4 $$ i_{t}= \sigma(W_{xi}x_{t}+W_{hi}h_{t-1}+W_{ci}c_{t-1}+b_{i})  $$

5 $$ f_{t}= \sigma(W_{xf}x_{t}+W_{hf}h_{t-1}+W_{cf}c_{t-1}+b_{f})  $$

6 $$ c_{t}=f_{t}c_{t-1} + i_{t}tanh(W_{xc}x_{t} + W_{hc}h_{l-1} + b_{c})  $$

7 $$ o_{t}= \sigma(W_{xo}x_{t} + W_{ho}h_{t-1} + W_{co}c_{t} + b_{o})  $$

8 $$ h_{t}= o_{t}tanh(c_{t})  $$

where *σ* denotes the logistic sigmoid function, *tanh* is the hyperbolic tangent activation function, and all weights (*W*s) and biases (*b*s) make up the parameter set (*θ*^*LSTM*^) of the LSTM layer. More details about the LSTM can be referred to in [32]. In sequence labelling tasks, it is better to be able to process both the past (from the left side) and the future (from the right side) context dependencies in the sequence. Therefore, another commonly used version of the LSTM is employed, called the Bidirectional LSTM (BiLSTM) [32, 37]. In the BiLSTM, for each word the forward LSTM captures the features from the left side and the backward LSTM captures the features from the right side. Each word effectively encodes information about the whole sentence.

#### Fully-Connected layer

The output of the BiLSTM layer at each time step *t*, obtained by concatenating the outputs of the forward and backward LSTMs $h_{t}=[h_{t}^{F};h_{t}^{B}]$, is mapped to a linear and fully-connected network layer using ReLU activation functions as follows:

9 $$ y_{t}= max(0,W_{t}h_{t}+b_{t})  $$

where all weights (*W*s) and biases (*b*s) make up the parameter set (*θ*^*F*^) of the fully-connected layer.

#### CRF layer

On the top of the fully-connected layer, a final CRF layer generates a sequence of labels for corresponding words. The CRF layer can learn the strong dependencies across output labels and come into the most likely sequence of the predicted tags [38].

### Transfer learning approach

The goal of cross-domain transfer in this study is to learn a sequence labelling model for triggers which transfers knowledge from a source domain to a related target domain.

#### TL model b

When the label sets of the source and target domains are inconsistent, including overlapping, it is treated as the case of the domains having completely different label sets in the basic idea of transfer learning. In this situation, the architecture of TL Model B is an extension of the basic BiLSTM-CRF model. And the source and target domains share the same input feature sets in the model. The TL Model B in Fig. 4 gives an overview of how to transfer parameters (*θ*s) of each neural network layer between both datasets within a certain range.

Let *s* and *t* represent the source domain and the target domain, respectively. And the parameter sets of each model layer *l* are $\theta _{s}^{l}$ and $\theta _{t}^{l}$ for the source and target domains, including the embedding layers ($\theta _{s}^{Emd}$ and $\theta _{t}^{Emd}$), the BiLSTM layers ($\theta _{s}^{LSTM}$ and $\theta _{t}^{LSTM}$), and the fully-connected layers ($\theta _{s}^{F}$ and $\theta _{t}^{F}$). The transfer learning process consists of learning the parameters ($\theta _{s}^{Emd}$, $\theta _{s}^{LSTM}$ and $\theta _{s}^{F}$) of a neural network on a source dataset, then transferring a part of them to another neural network and optimizing parameters ($\theta _{t}^{Emd}$, $\theta _{t}^{LSTM}$ and $\theta _{t}^{F}$) on a target dataset. In TL Model B, without the same label sets, only the parameters of the embedding and BiLSTM layers can be transferred and shared, as illustrated below:

10 $$ \theta_{s}^{Emd} = \theta_{s,shared}^{Emd}, \theta_{t}^{Emd} = \theta_{t,shared}^{Emd}, with\ \theta_{s,shared}^{Emd} \rightarrow \theta_{t,shared}^{Emd}  $$

11 $$ \theta_{s}^{LSTM} = \theta_{s,shared}^{LSTM}, \theta_{t}^{LSTM} = \theta_{t,shared}^{LSTM}, with\ \theta_{s,shared}^{LSTM} \rightarrow \theta_{t,shared}^{LSTM}  $$

where the subscript *shared* means the parameters that can be shared and transferred between the source and target domains. After training on the source domain, all the embedding and BiLSTM layer parameters, $\theta _{s}^{Emd}$ and $\theta _{s}^{LSTM}$, are mapped to initialize the parameters of the corresponding layers on the target dataset, $\theta _{t}^{Emd}$ and $\theta _{t}^{LSTM}$. Hence we have $\theta _{s,shared}^{Emd} \rightarrow \theta _{t,shared}^{Emd}$ and $\theta _{s,shared}^{LSTM} \rightarrow \theta _{t,shared}^{LSTM}$. It also means that the parameters of the fully-connected layer, $\theta _{s}^{F}$ and $\theta _{t}^{F}$, should be trained separately because of the inconsistent label sets.

#### TL model c

When with their own domain-dependent features, such as named entity type, the input feature sets of the source and target domains are inconsistent. The BiLSTM layers will have the different parameter dimensions and structures due to the different feature sets. Hence, the parameters of this layer cannot be shared neither. In this situation, the only parameters that can be transferred are from the embedding layer as shown in Eq. 12. More specifically, the shared parameters are those lookup tables trained for domain-independent features, *θ*_*s, shared*_={*TL*^*w*^,*TL*^*c*^,*TL*^*p*^,*TL*^*d*^}, where *TL*^*w*^ and *TL*^*d*^ are pre-trained. The TL Model C in Fig. 2 gives an overview of how to transfer the parameters between the neural network layers of both datasets.

12 $$  \theta_{s}^{Emd} \supset \theta_{s,shared}^{Emd}, \theta_{t}^{Emd} \supset \theta_{t,shared}^{Emd}, with\ \theta_{s,shared}^{Emd} \rightarrow \theta_{t,shared}^{Emd}  $$

#### Generalized tL model d (MLTrigNer): our transfer learning approach

This study uses the corpus with biomolecular trigger annotations as the source domain dataset and the corpus with multiple-level biomedical event triggers as the target domain dataset. Because of their inconsistent input feature and output label sets, we just can choose the TL Model C shown in Fig. 2 to build a trigger recognizer, without sharing the parameters of the fully-connected and BiLSTM layers. This ignores the information hidden in the overlapping features and labels. It is known in transfer learning that the more parameters are shared, the better generalization can be achieved in the target domain. For this purpose, we propose a generalized transfer learning architecture and approach to share as many parameters as possible to explore the transferability of each layer in a neural network, especially when the feature and label sets are overlapping.

As we discussed that parameters stand for the abstract features learned from a neural network. In the basic transfer learning architectures, TL Model B and C, the parameters are chosen to be transferred according to the network layers horizontally. When the label sets of the source and target domains are consistent, parameters from the upper (fully-connected) and middle (BiLSTM) layers can be transferred. Otherwise, when the label sets are inconsistent, the parameters of the whole upper layer closest to the output are discarded in TL Model B. Moreover, when the source and the target domains have inconsistent extracted feature sets, the parameters of the whole middle layer should be discarded in TL Model C. After careful study of the lower (embedding) layer of TL Model C, we find out that all these parameters learned from the source domain can be split into two parts: a source-specific part and a source-target-shared part. Correspondingly, the parameters of the target domain also can be split into two parts: a target-specific part and a source-target-shared part. This kind of divide is vertical within a network layer, and the source-target-shared part of the parameters can transfer the information carried by the overlapping of feature and label sets in the middle and upper layers. The main benefit is that we can include more domain-dependent features in the lower layer. For instance, in our trigger recognition task, there is a different and richer named entity type feature set in the target domain.

Figure 3 shows how we generalize the basic transfer learning approach to share as many parameters as possible. As mentioned, the parameters are split into two parts, domain-specific and domain-shared parameters:

13 $$  \theta_{s}^{l}= \theta_{s,speccific}^{l} + \theta_{s,shared}^{l}, \theta_{t}^{l}= \theta_{t,speccific}^{l} + \theta_{t,shared}^{l}  $$

where $\theta _{s,shared}^{l}$ and $\theta _{t,shared}^{l}$ are the parameters shared and mapped through the transfer learning in each layer *l*, and the domain specific parameters $\theta _{s,specific}^{l}$ and $\theta _{t,specific}^{l}$ are trained for each domain exclusively.

The degree of parameters to be transferred from the source network to the target network is determined according to the overlapping degrees of the input feature and output label sets between the source and target domains. Figure 3 shows the parameter sharing situation of the MLTrigNer. In general, suppose $\{x_{1}^{l}, x_{2}^{l},..., x_{j}^{l},...\}$ are the inputs of each layer *l*, $\{y_{1}^{l}, y_{2}^{l},..., y_{j}^{l},...\}$ are the outputs, and parameters *θ* of this layer are all weights (*W*^*l*^s) and biases (*b*^*l*^s). Since parameters can be divided into the domain-shared and domain-specific parts, their connected inputs and outputs can also be divided accordingly.

For the middle layers, such as the BiLSTM layers, of the source and target networks in Fig. 3, they have domain-specific and shared inputs of feature embedding vectors as $[x_{specific}^{l}, x_{shared}^{l}]$. Hence the corresponding domain-specific and shared connection weights for each output $y_{j}^{l}$ are $[W_{j,specific}^{l}, W_{j,shared}^{l}]$, and each output $y_{j}^{l}$ has its own bias $b_{j}^{l}$. The shared parameters in Eq. 13, $\theta _{s,shared}^{l}$ and $\theta _{t,shared}^{l}$, are $\{W_{shared}^{l}, b^{l}\}$. We can obtain each output $y_{j}^{l}$ as follows:

14 $$ \begin{aligned} y_{j}^{l} = active\_function & \left(\left[\left(W_{j,specific}^{l}\right)^{\mathrm{T}}, \left(W_{j,shared}^{l}\right)^{\mathrm{T}}\right]\right.\\ &\left.\left[ \begin{array}{c} x_{specific}^{l} \\ x_{shared}^{l} \end{array} \right] + b_{j}^{l} \right) \end{aligned}  $$

For the upper layers, such as the fully-connected layers, of the source and target networks in Fig. 3, they have domain-specific and shared label outputs as $[y_{specific}^{l}, y_{shared}^{l}]$. Hence the domain-specific and shared parameters for the corresponding outputs are $\{W_{j,specific}^{l}, b_{j,specific}^{l}\}$ and $\{W_{j,shared}^{l}, b_{j,shared}^{l}\}$, respectively. The shared parameters in Eq. 13, $\theta _{s,shared}^{l}$ and $\theta _{t,shared}^{l}$, are $\{W_{shared}^{l}, b_{shared}^{l}\}$. We can obtain each domain-specific output $y_{j,specific}^{l}$ and shared output $y_{j,share}^{l}$ as follows:

15 $$ {y_{j,specific}^{l} = active\_function\left(\left(W_{j,specific}^{l}\right)^{\mathrm{T}}x + b_{j,specific}^{l} \right)}  $$

16 $$ {y_{j,shared}^{l} = active\_function\left(\left(W_{j,shared}^{l}\right)^{\mathrm{T}}x + b_{j,shared}^{l} \right)}  $$

If the feature sets are the exactly same on both domains, there are no source-specific and target-specific parts of the parameters for the BiLSTM layers, $\theta _{s,specific}^{LSTM}= \emptyset $, $\theta _{t,specific}^{LSTM}= \emptyset $. Moreover, under this circumstance, if the label sets are completely different from each other on both domains, there are no source-target-shared parameters for the fully-connected layer, $\theta _{s,shared}^{F}=\theta _{t,shared}^{F}= \emptyset $, which is the TL Model B. On the other hand, if the label sets and the feature sets are inconsistent, we have $\theta _{s,shared}^{LSTM}=\theta _{t,shared}^{LSTM}= \emptyset $ and $\theta _{s,shared}^{F}=\theta _{t,shared}^{F}= \emptyset $, which is the TL Model C.

The training takes place over the following three main phases. First, the network is trained on the dataset from the source domain. Both $\theta _{s,specific}^{l}$ and $\theta _{s,shared}^{l}$ are learned. Then the shared parameters of each layer are transferred to the target domain, $\theta _{s,shared}^{l} \rightarrow \theta _{t,shared}^{l}$, to initialize the corresponding parts of the target model parameters. Finally, the network is trained on the dataset from the target domain. Both $\theta _{t,specific}^{l}$ and $\theta _{t,shared}^{l}$ are tuned and optimized.

## Abbreviations

BiLSTM-CRF: Bidirectional LSTM-CRF model
BiLSTM: Bidirectional LSTM
CNN: Convolution neural network
CRF: Conditional random field
DNN: Deep neural network
EPI: Epigenetics and post-translational modifications
LSTM: Long short term Memory
ML: Machine learning
MLTrigNer: Multiple-level trigger recogNizer system
NER: Named entity recognition
NLP: Natural language processing
POS: Part-of-speech
RNN: Recurrent neural network
ST: Shared task
SVM: Support vector machine
TL: Transfer learning
TM: Text mining
